# Hospital and Institutional News

**Published:** 1919-01-25

**Authors:** 


### HOSPITAL AND INSTITUTIONAL NEWS.

### DISPENSING MEDICINES IN THE ARMY.

The Army Council Instruction regarding the classification and employment of pharmacists and dispensers has now been issued. For the first time, dispensing in our army is to be conducted by pharmacists and dispensers who have recognised civil qualifications or can show proof of having had adequate training. The proposals are that there shall be three classes recognised—(1) Superintending Pharmacists; (2) Pharmacists holding the pharmaceutical qualification of Great Britain or Ireland; (3) Dispensers: (*a*) who have diplomas as assistants of the Society of Apothecaries, or (*b*) have passed the R.A.M.C. examinations for dispensers. Perhaps the most important point gained by the Pharmaceutical Society is that in all hospitals of 100 beds or over a superintending pharmacist will be in charge of the dispensing under the administrative control of the Medical Superintendent. Other persons engaged in dispensing may be pharmacists or dispensers. It is also stipulated that, although a hospital may have *less* than 100 beds, if the number of out-patients, where such attend, warrants it, a superintending pharmacist will be in charge of the dispensary, provided that the War Office are satisfied that this is desirable. So far, no rank higher than Staff Sergeant is to be granted (this to superintending pharmacists), but in all probability, as the scheme develops, commissioned rank will be granted to superintending pharmacists.

### ARMY EDUCATION AND MILITARY HOSPITALS.

The scheme of the Army Council to provide classes for officers and men still in the Army to prepare them for their return to civil life can be studied in the arrangements now made at the 3rd London General Hospital, Wandsworth. They are described in the New Year number of the hospital's *Gazette*. A whole-time education officer has been appointed to organise classes in general education and in technical and commercial subjects. Once a reasonable number of officers and men make a request for instruction in a particular subject, a class will be arranged for them. Modern languages (French, Italian, and Spanish are mentioned), as well as book-keeping, shorthand, mathematics, and music will be taught, and practical science is another subject which has been arranged for. The hospital education officer hopes also to prove a centre of information "on all matters connected with careers." The education officer is Captain T. B. Wheeler, and the following matter is worth quotation: "Under the Ministry of Labour scheme (officers' university and technical classes) arrangements exist by which certain officers, and in certain circumstances other ranks, may proceed to centres of instruction for university, technical, and business training, retaining their army pay and allowances." It will be interesting to watch the progress of this and kindred schemes. Many men whose education was interrupted by the war are impatient at the thought of returning to study, but its value to them will consist in opening a career of which the limit of income will not be three or four hundred a year.

### WILL BIRMINGHAM EXPLAIN?

Birmingham Corporation recently advertised for a Medical Officer to the Pensions Hospital, and expressed preference for "Senior men having recent hospital experience." "R.A.M.C." states in the *Times* that among the candidates were a lieutenant-colonel, a major, and three captains, Royal Army Medical Corps, all with war service. If the range of choice extended no further, Birmingham had here five officers fulfilling the main condition. But to the surprise and disgust of applicants the appointment was given to "a young recently-qualified man who has never served his country." An explanation appears to be due from the metropolis of the Midlands. If fully-qualified men who have made sacrifices for their country are treated in this cavalier manner it is a poor look-out for the lay officer, whose claims to consideration by the authorities of institutions has recently been under discussion in The Hospital.

### STUDY OF MENTAL AILMENTS

A drastic amendment of the Lunacy Acts and the Mental Deficiency Act is to be urged when the Visiting Committees of the Asylums in England and Wales meet in Conference in London Guildhall on February 5. The Acts in question are described as "effete" by the Committee which was appointed to report by the Conference last October. Presumably as a concession to feelings, in the sense that the word "workhouse" is being banished in many parts of the country, the terms "lunatic," "lunacy," and "asylum," it is proposed, shall be cast on the scrap-heap of inappropriate descriptions. But more far-reaching and important is the recommendation that local authorities should establish special mental hospitals for the treatment of incipient and actual mental disease, such institutions to comprise both in-patient and out-patient departments. It is further laid down that in connection with every medical school a special mental hospital should be established in which proper facilities are provided for research, teaching, and training; and that a medical officer entrusted with the care and treatment of the mentally afflicted should be required within two years of appointment to hold a recognised diploma showing that he is specially skilled in the science of the cure of mental ailments. Other important provisions are laid down, but sufficient has been summarised to show that a considerable awakening is taking place in a branch of medical science which has not hitherto been particularly popular or remunerative.

### YOUTH AND THE HIDDEN PLAGUE.

How can a knowledge of the frightful disasters that follow in the path of venereal disease be brought home to the young? The "hush" tactics of the past must come to an end. Everyone in a collective capacity agrees on that point, but individually all are cowards. It seems impossible to overcome the false modesty that exists among parents and religious teachers on this forbidding but not forbidden subject. Then how is youth and innocence to be warned of the dangers? Sir Robert Armstrong-Jones, Lecturer on Psychological Medicine to St. Bartholomew's Hospital, hints at a way. In his lecture at Gresham College on venereal disease and its prevention Sir Robert suggested that the new Education Act provides the authorities with an opening not previously in existence. Children of the masses have hitherto left school about fourteen; soon they are to continue compulsorily their studies up to sixteen. Why should not the State step in where guardians and religious leaders have feared to tread, and make it compulsory during the later years that a knowledge of hidden dangers should be imparted to these adolescents as part of the continuation education? It is good that the minds of the young should be trained, but it is much more important that they should learn how to protect their bodies against one of the most awful scourges that afflicts poor humanity.

### WOMEN AND METHYLATED SPIRITS.

The drug habit is not the only vice that has sprung into prominence during this era of dear and diluted intoxicants. Methylated spirit drinking is on the increase. So much so that the Liverpool licensing justices have been moved to petition the Home Office to take measures to check the evil. Like drug doping, the consumption of methylated spirit is far from being a new craving, but in the past, perhaps, its wretched victims have had to be sought, for the most part, among men. Women are now the great culprits. Mr. Harrison Jones, Chairman of the bench, said women of the poorer classes, in order to satisfy their desire, visited oil paint shops to buy methylated spirits prepared for commercial use, assuring the shopkeepers that they wanted it for mixing with paint. As the spirit, which is seven times stronger than ordinary whisky, contains paraffin and other disagreeable mixtures to make it unpalatable, one would think the stomach of either man or woman would rebel against it. But it has the power to stupefy, and everything else is forgiven. Needless to say, its effect is terrible physically and morally; and insanity is the end. If anything, this is a more serious evil than the purchase of drugs, but the resources of the Home Secretary are wide and powerful.

### THE LATE MR. MOULD.

The death has occurred of Mr. William Mould, M.R.C.S., who before his retirement was a well-known asylum superintendent with a long and varied experience. He was born in 1835 on the land which his family had long cultivated, and after being apprenticed to a Birmingham practitioner, he qualified in 1858. He then went temporarily to Sir Charles Hasting's private asylum at Droitwich, and removed from there to a post on the staff of the Birmingham asylum. He quickly became house surgeon to the Lancashire County Asylum at Prestwich with a salary of £800 a year and emoluments. On this he is said to have kept a horse and hunted with the harriers. Through the friends of his then chief, Dr. Holland, Mr. Mould became acquainted with a circle of Manchester doctors, and among them was the chairman of the Manchester Royal Infirmary, and after refusing the post of Medical Superintendent to the asylum now known as Cheadle Royal Asylum, he accepted it when it was offered to him again in 1862. There were then only sixty patients, but after forty years of hard work under Mr. Mould the number rose to 300. He was twice married. When he retired, he lived at Colwyn Bay, and his friends still remember his wit and humour, and the stories that they always associated with him.

### PRESENTATION TO A HOSPITAL SECRETARY.

A presentation has now been made to Mr. Walton Davies who retired after 21 years' services in August last from the secretaryship of the Preston Royal Infirmary, a post which he combined with the work of a chartered accountant. The presentation, which was made by Dr. R. C. Brown, a veteran in hospital work, consisted of a silver gilt claret jug of 1837. This was a testimonial of regard for Mr. Davies from the Board and the medical staff. In The Hospital of September 28 last, we gave some of Mr. Davies' recollections in regard to the changes which had taken place during his administration.

### THE NEW SECRETARY AT PRESTON.

These changes have not come to an end with his successor, Mr. John Gibson. It is only since September that the Secretary's office has been situated in the Royal Infirmary itself. The war work was previously carried on at 5 Winckley Street. The new out-patients' clinics in the town for the treatment of venereal disease have had three months' experience behind them, and another new departure is the Board's decision to appoint a teacher of massage to instruct the sisters for the June examination of the I.S.T.M.—a decision which may lead to the establishment of a permanent massage department. The uniform system of Hospital Accounts has also been adopted recently. Mr. Gibson, who is in his thirty-second year, was educated at Kendal Grammar School. After becoming proficient in commercial subjects, he became a clerk in a firm of solicitors, and for five years was its cashier. His administrative experience began with his appointment as chief clerk to the Borough Treasurer of Kendal, and the accounts of which he then had charge included those of the local infectious diseases hospitals. Mr. Gibson gave much of his spare time to organising voluntary agencies, and became, among other things, honorary secretary and treasurer to the Kendal Y.M.C.A. This gave him his first opportunity to convert a debt into a balance-in-hand. As a matter of detail we may record that in 1917 he was associated with the Kendal Local Tribunal, a word which is beginning to carry a flavour of the past.

### NORTH STAFFORDSHIRE'S AMBITION.

THE annual meeting revealed a gratifying state of affairs at the North Staffordshire Infirmary, and a laudable desire to do even better in the future. Progress was reported in nearly every department. In-patients had increased by 144, the total being 2,947, and out-patients by 1,000, with a total of 20,456. Finance was a particularly pleasing feature. There was actually a surplus of £2,846 on the year's working, much of the credit for this being due to the working classes. It is wonderful, too, in these days of continually rising prices to learn that in the housekeeping department it had been possible to effect a saving of over £1,000, while providing for the treatment of over 1,000 more patients. It would be interesting to learn from the housekeeper and the management just how this miracle has been accomplished. No really healthy institution is ever happy unless it is making progress. North Staffordshire Infirmary sighs for greater scope, and it is pretty safe to prophesy that this will be forthcoming. For the district takes great pride in the institution. There is talk of providing 250 more beds in order to meet the demands of the waiting list. This would mean additions to the permanent buildings. If North Staffordshire wishes to make a "thank-offering" in celebration of peace here is a deserving object ready to hand. The Mayor in his speech acknowledged the claims of the Infirmary, and while in no position to make pledges, significantly stated that he "believed it was in the minds of the people that the money raised should be devoted to beneficent purposes for those in need of help."

### THE FUTURE AT HARTSHILL.

IT must be encouraging for Mr. W. Stevenson, who succeeded Major Basil Rhodes as secretary and house-governor to the North Staffordshire Infirmary at Hartshill, that the latest Saturday Fund collections are £790 more than they were in 1917. Meantime, he has been busy, and addressed many meetings in the district, so that he has done his best to continue the personal relations between the hospital and everyone in its district, on which its success and popularity have been built in the past. In methods of treatment pains have been taken to make the hospital an up-to-date orthopædic centre. The proposed Ministry of Health will be the next change to which the voluntary hospitals will have to adapt themselves, and we can only repeat that happy will that hospital be which is so equipped as to depend least upon official help and so to be least burdened by official interference.

### BROXBOURNE COTTAGE HOSPITAL SCHEME.

A SCHEME is on foot to establish a cottage hospital at Broxbourne, but the success of the plan depends on whether two other Hertfordshire villages, namely, Hoddesdon and Wormly, will combine with Broxbourne to carry it through. The scheme would be the local war memorial, and its only rival is a plan to make additions to Broxbourne church.

### HIGHER SALARIES AT BEDFORD HOSPITAL.

WE are glad to record that the Board of the Bedford County Hospital has decided to raise the salary of the secretary by £100 a year, and that of the dispenser and radiographer by £70 a year. These increases began on January 1 and reflect the public spirit of the hospital's chairman, Mr. J. Arnold Whitchurch, and of his colleagues on the Board.

### CRITICAL MOMENT AT NORWICH.

SEVERAL peace offerings have been received at the Norfolk and Norwich Hospital, and they have been sufficient to be invested, while the bed maintenance fund, which was established four years ago, has given birth to an organisation which, it is hoped, may prove permanent. The various firms which have supported the fund are now to have a representative upon the board of management. Indeed it is necessary that the fund should grow, for the board has agreed to accommodate twenty discharged soldiers, Norfolk men, and the children's ward is to be re-opened. Besides this, many delayed repairs cannot now be further postponed, and their cost is expected to run into four figures. The railwaymen in the Northern division are coming to the rescue, but the moment is critical, and the future uncertain.

### INFLUENZA CASES AND FEVER HOSPITAL.

THE Town Council of Boston in Devonshire recently discussed the possibility of admitting influenza and pneumonia cases into the local fever hospital. Dr. South stated that such cases, in times of epidemic, should be admitted to the fever hospital, and that it was a shame that people all over the country had been allowed to die for want of institutional treatment. Boston Hospital, he added, was useless for such cases, but the difficulty in sending them to a fever hospital was the risk that scarlet fever cases might also be there. In these circumstances a separate block would have been essential, but where there are no scarlet fever cases medical men should know that patients suffering from influenza and pneumonia could be admitted. It was agreed that the doctors should be informed.

### DEATH OF MR. W. F. CHALKLEY.

AFTER forty years' service on the board of delegates of the Hospital Saturday Fund, Mr. Walter Frederick Chalkley lately died after three months' illness. A railway worker for most of his life, in 1879 he became the delegate of the Amalgamated Society of Railway Servants. The local committee, of which he subsequently became chairman in 1905, presented him with an address and a portrait to commemorate twenty-five years' work. From 1909-11 he was chairman of the council, and after that he was an *ex-officio* member of the executive committee.

### SOUTH WALES BENEFACTORS.

THE Prince of Wales' Hospital for Limbless Sailors and Soldiers is finding no lack of generous supporters throughout South Wales. Gifts have been flowing in steadily to the endowment fund, among the most notable of recent ones being sums of £1,050 from Mr. W. T. Farr, Mumbles; Mr. Henry G. Lewis, Porthkerry; and Mr. E. Plisson, Cardiff; while an anonymous donor has undertaken to subscribe one hundred guineas a year for ten years. The Celtic Collieries, Limited, and the United National Collieries, Limited, are also among the subscribers, the former with £150 and the latter with £105. Cheques for one hundred guineas have been received from Mr. James Miles, Cardiff, and Mr. Sydney L. Gregor, Westcross, and one from Lord Colum Crichton Stuart for £100.

### A HOSPITAL SPARRING MATCH.

The men patients of the Royal Hospital for Incurables, Putney Heath, recently met for their annual smoking concert, and were entertained by music, songs, and a sparring match between Dr. John Gay, the medical officer, and the Secretary. This amateur display consisted of ten rounds, with gloves before a referee. After the evening's programme was over, Mr. Charles Stopp, who took the chair, gave a friendly public welcome to Mr. Henry Luscombe, who has lately been appointed steward. The patients look forward to these occasions not only for the fun of the evenings and the music, but for the good things, including tobacco, which are given to them by the hospital's friends.

### A WOMAN'S LONG SERVICE IN THE POOR LAW.

Miss Bird has just completed twenty years' service as assistant relieving officer under the Southwark Board of Guardians, and the guardians have recorded their satisfaction at her work. They have further decided, with the approval of the Local Government Board, to alter the title of her office to "lady relieving officer." Miss Bird, we believe, is the only woman, in London, at any rate, who has held an office of this kind continuously under one authority for twenty years. Her duties have been closely associated with the hundreds of young children who have unfortunately come under the care of the guardians, and she has dealt with them as a woman of wide sympathies.

### THE DECREASE IN THE SICK POOR.

A request from the district medical officers of the Southwark Union that they shall be granted an increase of remuneration by way of war bonus or gratuity has brought to light in a startling fashion the great decrease in the number of sick poor seeking assistance. The request was based not only on account of the increased cost of living and the decreased spending value of money, but also because of the increased work by reason of attendance on the wives and children of interned persons, and by absence on service of many of their colleagues. A return was prepared by the clerk, Mr. A. P. S. Smith, showing the number of cases dealt with by the district medical officers in the years ending September 30, 1914 and 1918. The result showed that in the four years the number of poor sick seeking aid has fallen nearly one-half in number. Under these circumstances the guardians had no option except to defer for six months further consideration of the medical officers' request.

### PERPETUATING A WARTIME AGENCY.

In South Buckinghamshire an attempt is being made to persuade the women who have been regularly collecting for the Red Cross by gathering penny a week subscriptions in the towns and villages to carry on the work on behalf of the scheme for building a War Memorial Hospital at High Wycombe, the industrial centre of the county. A site has been offered, and some £4,000 has been given by a few subscribers. A total sum of £15,000 is required. So many useful organisations are likely to lapse through want of foresight, that it is good to record every attempt made to perpetuate them for the Voluntary hospital.

### CHILD WELFARE IN CAMBERWELL.

A step in the right direction has at last been taken by the Camberwell Borough Council, who have decided to contribute £50 towards the funds of the Camberwell and Dulwich Homes at De Crespigny Park, under the maternity and child welfare scheme. This is the first donation of the kind made by the Council, though neighbouring boroughs long ago saw the wisdom of assisting this work. The proposal, however, did not meet with the full approbation of the councillors, one of whom thought that the guardians, and not the council, were the people who should look after expectant mothers, and, later on, their offspring. Happily such a view did not meet with much support. The home is pleasantly situated, and has accommodation for ten mothers and six babies. Two trained maternity nurses are in residence, and a doctor is also provided.

### THIS WEEK'S DRUG MARKET.

There is not the slightest improvement in business on the drug and chemical markets; if anything it is quieter than ever, and buyers show no signs of being tired of waiting for prices to go down. On the other hand, there are indications that their patience may be rewarded in due course, for although there is as yet nothing which promises a general slump, several parcels of various drugs and chemicals have recently been hurried on to the market and disposed of at bargain prices. After going to press last week a large quantity of assorted drugs was offered in public auction, but the bidding was extremely slow and the bulk of the offerings were bought in. Where sales were effected they were generally at lower prices, while in several instances lots were sold at extraordinarily low prices. Honey, saccharine, and sugar of milk have all been sold at substantially reduced figures. Eucalyptus oil is in good supply, and is being offered at lower rates. It is, however, of little practical utility to mention instances, for in a large number of cases prices are merely nominal. The advice offered in recent numbers—to refrain from buying as far as possible—can again be repeated.

